# Risk negotiation: a framework for One Health risk analysis 

**DOI:** 10.2471/BLT.23.290672

**Published:** 2024-05-08

**Authors:** Monika Ehling-Schulz, Matthias Filter, Jakob Zinsstag, Konstantinos Koutsoumanis, Mariem Ellouze, Josef Teichmann, Angelika Hilbeck, Mauro Tonolla, Danai Etter, Katharina Stärk, Martin Wiedmann, Sophia Johler

**Affiliations:** a Institute for Microbiology, University of Veterinary Medicine Vienna, Vienna, Austria.; b German Federal Institute for Risk Assessment (BfR), Berlin, Germany.; c Human and Animal Health Unit, Swiss Tropical and Public Health Institute, Allschwil, Switzerland.; d Department of Food Science and Technology, Aristotle University of Thessaloniki, Thessaloniki, Greece.; e Department of Digital Food Safety, Nestlé Research, Lausanne, Switzerland.; f Department of Mathematics, Federal Institute of Technology Zurich, Zurich, Switzerland.; g Institute of Integrative Biology, Federal Institute of Technology Zurich, Zurich, Switzerland.; h Institute of Microbiology, University of Applied Sciences and Arts of Southern Switzerland, Mendrisio, Switzerland.; i Institute for Food Safety and Hygiene, Vetsuisse Faculty, University of Zurich, Winterthurerstrasse 272, 8057 Zurich, Switzerland.; j Federal Food Safety and Veterinary Office, Bern, Switzerland.; k Department of Food Science, Cornell University, Ithaca, United States of America.

The world faces global health risks that need to be effectively addressed in integrated, participatory efforts.^1^^,^^2^ However, risk analysis frameworks do not account for the complex nature of systems that span multiple sectors or disciplines.^2^^,^^3^

We propose the participatory and interdisciplinary concept of risk negotiation to transform the way we tackle global health challenges such as pandemics, physical and mental health inequities, environmental problems and food security. To allow such risk analysis, we need to recognize the value of risks and trade-offs and negotiate them with stakeholder groups representing different disciplines and sectors. This approach becomes feasible through recent technological breakthroughs such as artificial intelligence-assisted multiagent negotiations or large language models. These models are accessible, hold promise in negotiating agreements^4^ and can be used to accommodate the complexity of real-world decision-making.

## A risk negotiation framework

**Fig. 1:**
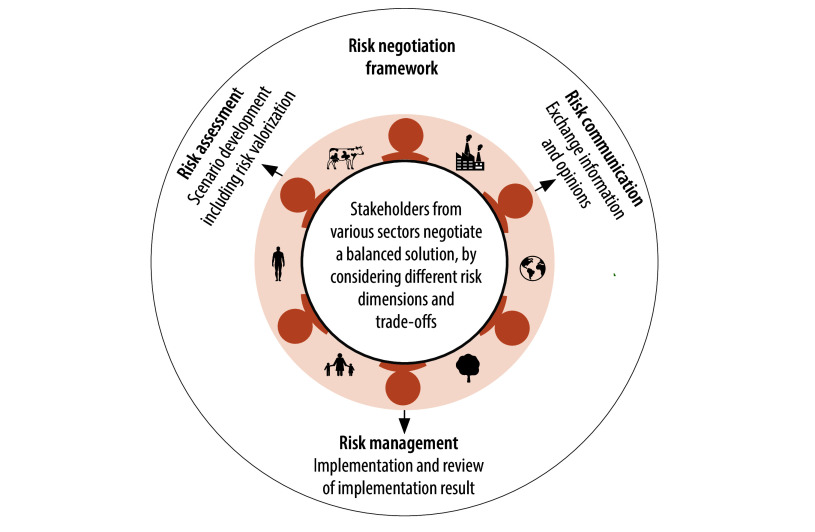
Risk negotiation framework

At the heart of our concept is a simple yet profound idea: negotiating risks (Fig. 1). Integrating risk negotiation in risk analysis frameworks enables a holistic assessment of health risks. This participatory approach allows dialogue between representatives of different stakeholder groups and thus enhances cooperation between them. Such integration empowers stakeholders to reach a balanced solution to problems that considers multiple risk dimensions and trade-offs, allowing an effective management of health risks. We propose to implement this negotiation-centred risk analysis framework in six steps, whereby steps 2–6 could be supported by artificial intelligence tools. 

First, establishing a stakeholder representatives roundtable, whereby the lead agency or a multisectoral team map and identify stakeholders for a particular problem.^5^

Second, problem formulation, whereby the roundtable collectively formulates a problem (or multiple subproblems) and identifies one or more lead disciplines for risk assessment and valuation. Problem formulation can be aided by conversational large language models such as ChatGPT (OpenAI, San Francisco, United States of America) or Perplexity.ai. (Perplexity, San Francisco, USA). Potential data privacy concerns could be overcome by containing the large language models in protected environments, for example using open source large language models from Hugging Face (Hugging Face, New York, USA) inside frameworks such as Ollama (Ollama, Toronto, Canada).

Third, risk assessment and valuation. Risk assessors undertake a multidimensional, evidence-based risk assessment and consider different action options and trade-off analyses. The process results in valuation of the risks. By analysing vast data sets and employing advanced predictive analytics, artificial intelligence can enhance holistic risk assessment accuracy and facilitate cost estimation of the different risks. Nevertheless, to avoid inherent bias in the training sets used to train artificial intelligence models that could lead to marginalization of minority perspectives, human supervision is needed. 

Fourth, risk negotiation. The stakeholder representatives negotiate a balanced solution and recommend an action option within a given time frame. From a computer science perspective, this risk negotiation process can be treated as a multiagent game involving cooperation and competition that can be supported by artificial intelligence. Recent breakthroughs have enabled artificial intelligence solutions for negotiating and reaching agreements by multiple actors^6^^,^^7^ that can facilitate and accelerate negotiation-based risk analysis frameworks. For example, artificial intelligence can: (i) serve as an artificial stakeholder or mediator; (ii) suggest decisions by solving a virtual version of the decision-making process using solution algorithms for multiagent games,^6^^,^^7^ thus identifying equilibria of maximized benefits and minimized risks; and/or (iii) simulate the consequences of decisions and devise actionable solutions by using a human-in-the-loop approach in which human judgement is integrated to enhance the process and supervise outcomes. Different large language models for coding, simulating and solving will facilitate these applications.⁠ Software solutions such as AutoGen Studio (Microsoft, Redmond, USA) that allow to implement these processes are already available and provide proof-of-concept. Yet, as artificial intelligence tools might struggle to sufficiently integrate cultural and ethical considerations, human supervision is again crucial.

Fifth, communication and implementation. Roundtable participants inform their stakeholder groups about negotiation outcomes and implement the risk management strategies. Decisions, associated actions and their risks can be communicated and implemented using specialized large language models that govern the pipeline between the decision and its realization in the real world. For example, multimodal artificial intelligence tools such as ChatGPT or Google Gemini (Google LLC, Mountain View, USA) could aid in editing and visualizing text, and convert expert reports to short summaries accessible to lay audiences.

Sixth, outcome evaluation and risk re-negotiation. The stakeholders evaluate the risks and benefits to their sector and communicate the negotiation outcome via their representatives to the roundtable, which can initiate a renegotiation or a new round of risk analysis. Artificial intelligence can support the monitoring and reporting process by gathering information automatically via artificial intelligence agents or analysing them, as demonstrated by the project ARIES.^8^ Through ARIES, an artificial intelligent modeller builds agents, connects them into a flow network and creates models for each agent and connection. ARIES can be used to track and forecast progress towards achieving the sustainable development goals (SDGs). Furthermore, artificial intelligence can assist negotiations as in step 4.

## Application in One Health

Food safety is a prominent feature in SDG 2^9^ and is a classical One Health issue, intertwined with food security and socioeconomic growth. Each year, there are an estimated 600 million cases of foodborne disease, resulting in a global burden of 33 million disability-adjusted life years (DALYs) and 420 000 premature deaths.^10^ Food safety management systems to mitigate foodborne risks rely on risk analysis.^11^ In many geographical contexts, risk managers formulate a question and prompt a scientific risk assessment that is conducted for a single discipline,^12^ despite the need for an interdisciplinary, participatory approach to risk analysis. The World Health Organization called on governments, industry, consumers and civil society to improve food safety systems as part of its 2022–2030 strategy.^9^ Restructuring the food safety risk analysis framework around risk negotiation could help balance food safety, food security and sustainability. For instance, while a zero-tolerance strategy against a foodborne hazard might reduce exposure to potentially contaminated food, food prices and food waste would likely increase, which would harm food security and contribute to health inequalities, without reaching zero risk. The introduction of risk valuation-based risk negotiation would promote interdisciplinary risk assessments and the evaluation of trade-offs for different action options. We propose enabling stakeholders to negotiate an acceptable solution based on consensus criteria, a process that could be expedited by recently developed artificial intelligence-powered solutions. Using transparent, holistic and ethically grounded agent-based frameworks would foster consensus generation and support the communication and the implementation of outcomes, followed by evaluation and renegotiation processes.

## Implementation challenges

A central challenge in implementing a risk negotiation-centred risk analysis approach lies in the identification of appropriate stakeholder groups and representatives. Careful stakeholder identification and mapping will be key, and each outcome should be evaluated considering a critical review of the composition of the stakeholder roundtable. Completeness of the identified stakeholders is a critical factor for success. Facilitating knowledge sharing within the stakeholder group is as important as sharing perspectives. 

Another challenge is the lack of a common currency (such as monetary value or DALYs) to weigh risks and benefits between sectors. Our concept allows stakeholders to balance risks expressed in different units (such as risk of disease measured using DALYs versus biodiversity losses measured using the biodiversity index), by defining acceptable limits for each risk. Artificial intelligence-assisted multiagent negotiation can help find these balances if agents can agree on risk exchanges.^13^ Using transparent, self-critical and ethically grounded agent-based frameworks will also help to address certain risks linked to artificial intelligence technologies such as the risks of bias and lack of transparency.

Another difficulty lies in balancing stakeholder interests under time and information constraints, as during the coronavirus disease 2019 (COVID-19) pandemic. Although the need for the integration of social norms and ethical considerations has prevented the implementation of balanced risk assessment in the past, recent artificial intelligence breakthroughs may enable balanced and accelerated decision-making processes. Artificial intelligence-based negotiation tools must be adapted to the specific decision context because much of our perception of risk is deep-rooted in culture and beliefs. To reach a consensus, the social norms that define the payoff functions of multiagent risk strategies must be negotiated.^13^ Open source implementations already exist that allow for integration of general social norms and ethical considerations into multiagent negotiation frameworks as a default.

## Added value

Negotiation-based risk analysis frameworks assisted by artificial intelligence can transform traditional, discipline-centred, sectoral risk assessments into participatory and holistic processes. For instance, risk negotiation could foster the operationalization of One Health as an enabler for the integration of human, animal and environmental health trade-offs in one risk analysis framework. As outlined in the One Health Theory of Change,^1^ implementation of One Health approaches facilitates prevention, prediction, detection and response to health threats. Risk negotiation could be instrumental in overcoming obstacles to the adoption of the One Health paradigm, which stem from the fragmentation of health sciences into subdisciplines. The integration of risk negotiation will help leverage the knowledge of different stakeholders and sectors or disciplines that an individual expert could not oversee alone. Risk negotiation could also transform risk communication: while risk communication is traditionally unidirectional (that is, from authorities to consumers) risk negotiation would stimulate stakeholder engagement throughout the process, thus increasing trust and community acceptance of risk mitigation actions. The proposed iterative process design increases the flexibility of risk management actions to allow adjustments based on evaluation of the outcomes. In addition, a risk negotiation-centred risk analysis framework would increase transparency and be conducive to conflict prevention and resolution as well as to the development of sustainable, equitable and inclusive solutions, as it is rooted in collaboration and co-development of solutions.

## Conclusion

The challenge of addressing complex health risks in today’s interdependent societies requires a transformation of traditional risk analysis frameworks. Integrating risk negotiation would empower stakeholders to engage in a participatory approach to balance different risk dimensions and trade-offs. Artificial intelligence technologies will support the negotiation of agreements between sectors and stakeholders in a fit-for-purpose timeframe. This approach requires stakeholder participation throughout the entire process, which will lead to increased trust and community acceptance of risk mitigation actions. In this regard, risk analysis frameworks centred on risk negotiation could foster transparency and increase global health security, equity and sustainability.
